# Comparative costs for critically ill patients with limited English proficiency versus English proficiency

**DOI:** 10.1371/journal.pone.0279126

**Published:** 2023-04-26

**Authors:** Amelia K. Barwise, James P. Moriarty, Jordan K. Rosedahl, Jalal Soleimani, Alberto Marquez, Timothy J. Weister, Ognjen Gajic, Bijan J. Borah

**Affiliations:** 1 Division of Pulmonary and Critical Care Medicine, Mayo Clinic, Rochester, Minnesota, United States of America; 2 Bioethics Research Program, Mayo Clinic, Rochester, Minnesota, United States of America; 3 Kern Center for the Science of Health Care Delivery, Mayo Clinic, Rochester, Minnesota, United States of America; 4 Department of Anesthesiology and Perioperative Medicine, Mayo Clinic, Rochester, Minnesota, United States of America; 5 Anesthesia Clinical Research Unit (ACRU), Department of Anesthesiology and Perioperative Medicine, Mayo Clinic, Rochester, Minnesota, United States of America; Yale University, UNITED STATES

## Abstract

**Objectives:**

To conduct comparative cost analysis of hospital care for critically ill patients with Limited English Proficiency (LEP) versus patients with English proficiency (controls).

**Patients and methods:**

We conducted a historical cohort study using propensity matching at Mayo Clinic Rochester, a quaternary care academic center. We included hospitalized patients who had at least one admission to ICU during a 10-year period between 1/1/2008-12/31/2017.

**Results:**

Due to substantial differences in baseline characteristics of the groups, propensity matching for the covariates age, sex, race, ethnicity, APACHE 3 score, and Charlson Comorbidity score was used, and we achieved the intended balance. The final cohort included 80,404 patients, 4,246 with LEP and 76,158 controls. Patients with LEP had higher costs during hospital admission to discharge, with a mean cost difference of $3861 (95% CI $822 to $6900, p = 0.013) and also higher costs during index ICU admission to hospital discharge, with a mean cost difference of $3166 (95% CI $231 to $6101, p = 0.035). A propensity matched cohort including only those that survived showed those with LEP had significantly greater mean costs for all outcomes. Sensitivity analysis revealed that international patients with LEP had significantly greater overall hospital costs of $9,240 than patients with LEP who resided in the US (95% CI $3341 to $15,140, p = 0.002).

**Conclusion:**

This is the first study to demonstrate significantly higher costs for patients with LEP experiencing a critical illness. The causes for this may be increased healthcare utilization secondary to communication deficiencies that impede timely decision making about care.

## Introduction

The number of people in the US who have Limited English Proficiency (LEP) continues to increase. According to the 2017 US Census, more than 64 million people aged 5 years and older speak a language other than English at home, and more than 8.5% of the US population can be classified as “speaking English less than very well” or with having LEP [1, 2]. Like the rest of the US population, this group of people is also aging, more likely to have medical co-morbidities, potentially increasing the likelihood they may end up being admitted to the ICU for treatment [3].

There are a large number of patient-related factors influencing ICU outcomes including acuity and severity of illness at presentation, as well as the presence of co- morbidities [4]. Other important patient characteristics that influence outcomes and lead to differences in recovery and survival include age, and demographic factors such as sex, ethnicity, and race [5–8]. Disparities in critical illness outcomes have been well documented related to race and ethnicity [5, 6, 9, 10]. Some of these disparities may relate to institutional differences in the settings and hospitals in which care was provided rather than individual patient factors [9, 10].

In the general inpatient setting, patients with LEP have longer hospital stays and increased rates of re-admission [11, 12]. In the ICU, the literature and our own work has highlighted prolonged use of life-support interventions and increased use of full code measures among patients with LEP who experience critical illness [13, 14].

Although only one quarter of hospitalized patients in the USA get admitted to the ICU, the costs of caring for ICU patients accounts for half of total hospital expenditure, estimated to be between $110 to $260 billion per year or approximately 1% of the United States’ gross domestic product [15, 16]. The demand for ICU care varies geographically but overall continues to increase, and costs continue to accelerate [17, 18]. Research here and abroad has shown that the cost of human resources required for prolonged bedside care, as well as the cost of interventions such as dialysis and mechanical ventilation, contribute to ICU costs [19, 20]. Other studies support these findings and also found costs correlated with assessments of severity of illness [21, 22].

Costs among racial and ethnic minority groups at end of life are higher for these minority groups partly related to greater use of life sustaining interventions [23]. Cost studies related to patients with LEP tend to focus on economic evaluations of interpreter services [24, 25]. However, no studies exist exploring the issue of cost for patients with LEP requiring critical illness care. The purpose of this study is to evaluate the costs of the ICU and total hospital stay for patients with LEP and compare the costs to those of English proficient (EP control) patients. We hypothesize that patients with LEP will have higher costs [14].

## Methods

We conducted a single center cohort study of hospitalized patients who had at least one admission to one of the ICUs at Mayo Clinic Rochester during a 10-year period between 1/1/2008-12/31/2017. Mayo Clinic is an academic quaternary care center with over 2000 inpatient beds allocated between two hospital campuses, St. Mary’s Hospital and Methodist Hospital. The institution has approximately 62,000 admissions annually. The hospital has approximately 220 ICU beds and 16,000 ICU admissions annually. Mayo Clinic, Rochester, MN is one of the leading healthcare institutions in the US with an established program for providing complex care to both domestic and international patients [26]. The study was approved by the Mayo Clinic Institutional Review Board (IRB) as an exempt study (IRB number 19–009625). The study is reported in accordance with the STROBE guidelines for observational studies [27]. The inclusion criteria were patients 18 years or older who were admitted to the ICU within the specified time period and who had provided prior research authorization and had, therefore, given prior consent to having their medical records reviewed for research purposes (per Minnesota statute). There was no contact with patients.

### Definition of limited English proficiency

Patients were classified as having LEP or not based on documented primary language other than English in the electronic medical record (EMR), consistent with a definition we have used in other publications [14].

### Definition of ICU admission

If a patient was admitted to ICU several times during a single hospitalization, the first admission to the ICU was considered the index ICU admission of that hospitalization, but all admissions were included in the cost analysis. For patients with multiple hospitalizations having an ICU stay, each hospitalization was included and considered independent from all other ICU hospitalizations of that patient.

### Demographic and clinical data collection

Data was abstracted using the Advanced Cohort Explorer (ACE), an electronic retrieval query database within Mayo Clinic’s Unified Data Platform (UDP) and DataMart. Validated searches were used to query the EMR. DataMart is an extensive data warehouse containing a near-real-time normalized replica of Mayo Clinic’s EMR. DataMart contains patient demographic characteristics, diagnoses, laboratory results, and clinical flow sheets, gathered from various sources within the institution. The data within DataMart has been validated and is reliable [28]. Medical complexity was assessed with the Charlson Comorbidity Index; this index reflects the number and severity of 19 predefined comorbid conditions (as identified by International Classification of Diseases, Ninth Revision codes), providing a weighted score of a patient’s comorbidities [29]. The Acute Physiology and Chronic Health Evaluation (APACHE) III score was calculated for each patient to assess illness severity 24 hours after admission to the ICU [30]. Additional demographic characteristics included were age, sex, race and ethnicity.

### Cost data collection

Cost data was collected from the Mayo Clinic Rochester Cost Data Warehouse (MCR-CDW) [31]. This database contains all billed services for patients seeking care at Mayo Clinic Rochester. These services are standardized using year specific Medicare reimbursement rates for professional services and multiplying billed charges by year specific department level cost-to-charge ratios as reported in the Medicare cost reports. All costs were inflation adjusted using the Gross Domestic Product Implicit Price Deflator. Costs are reported in 2018 US Dollars. Billed services from the MCR-CDW include date of service but not time of day the service took place. This prevents perfect differentiation of ICU costs and costs occurring on the general floor on the dates of ICU admission and discharge. Due to this limitation in the data, all costs occurring on dates of ICU admission and discharge were assumed to be ICU costs.

### Study outcomes

The primary study outcome was cost of the entire hospitalization. Secondary outcomes included costs from index ICU admission to hospital discharge, cost of all ICU admissions, and costs from post index ICU discharge to hospital discharge.

### Statistical analysis

To control for potential imbalance in patient characteristics between the LEP and control groups, propensity score matching was utilized. A one-to-one, nearest neighbor matching approach was used without replacement [32]. Common support was checked for model covariates. Covariates used in propensity score matching included age, sex, race, ethnicity, APACHE III score, and Charlson Comorbidity score. Balance in patient characteristics pre- and post-matching were compared using standardized differences. A standardized difference within -10% and +10% was considered to be balanced [33]. Costs were compared using differences in predicted mean cost following generalized linear modeling of cost as a function of LEP in the propensity-matched sample, with gamma distribution for cost and logarithmic link [34]. Robustness of study results were investigated analyzing various sensitivity analyses. Among the patients with LEP, costs were compared between those that were international vs. non- international patients. Additionally, a secondary propensity score matching analysis was performed only on patients that survived to discharge. All analyses were performed in Stata/MP 16.1.

## Results

A total of 4,246 LEP and 76,158 control patients were eligible for study inclusion. Patient characteristics in the pre- and post-matching samples are shown in Table 1. There was a large degree of imbalance pre-matching in patient characteristics based on standardized differences. Patient characteristics out of balance included age, race, ethnicity, and Charlson score. However, balance was achieved following propensity score matching with all included model covariates having standardized differences within the balance limits of ±10%.

**Table 1:** Baseline characteristics pre and post propensity matching.

	Pre matching					Post matching				
	Control		LEP			Control		LEP		
	Mean SD or		Mean SD or		Std Diff	Mean SD or		Mean SD or		Std Diff
	N = 99356 (%)		N = 5127 (%)		(%)†	N = 99356 (%)		N = 5127 (%)		(%)†
Age	62.5	17.4	58.3	18.5	23.4	57.2	18.4	58.3	18.5	-5.7
Female	42248	49.4	2184	49.5	-0.2	2182	49.4	2184	49.5	-0.1
Race										
White	97279	97.9	4366	85.2	47.5	4393	85.7	4366	85.2	7.7
Asian	258	0.3	244	4.8		179	3.5	244	4.8	
Black/African American	539	0.5	107	2.1		101	2.0	107	2.1	
Other	1134	1.1	329	6.4		383	7.5	329	6.4	
Unknown	148	0.1	81	1.6		71	1.4	81	1.6	
Ethnicity										
Non-Hispanic/Latino White	97257	97.9	4569	89.1	36.4	4492	87.6	4569	89.1	5.2
Hispanic/Latino	329	0.3	166	3.2		170	3.3	166	3.2	
Other	1772	1.8	392	7.6		465	9.1	392	7.6	
Apache3 Score	58.8	24.4	59.2	26.6	-1.4	58.6	26.3	59.2	26.6	-2.3
Charlson Score	5.3	3.5	4.7	3.4	18.0	4.5	3.4	4.7	3.4	-5.5
Ln pre index ICU costs	1.9	3.7	1.9	3.8	-1.5	2.0	3.8	1.9	3.8	2.4

^†^ Percentage values between -10% and +10% are considered in balance, and values outside that range are out of balance

Ln = natural Log

### Primary and secondary outcomes

Primary and secondary outcomes are shown in Table 2. Costs for patients with LEP from the complete hospitalization were $3,861 greater than that of controls (95% Confidence Interval (CI) $822 to $6,900, p = 0.013). Similar results were found for costs from index ICU admission to hospital discharge with a mean difference of $3,166 in costs (95% CI $231 to $6,101, p = 0.035). Costs of all ICU admissions were not statistically significant between patients with LEP and controls, (mean difference = $1670, 95% CI -$1034 to $4375, p = 0.226). Costs of post index ICU discharge to hospital discharge were also not statistically significant, (mean difference = $1812, 95% CI -$478 to $4103, p = 0.121). However, these results both show similar directionality to the primary outcome and the findings about costs from index ICU to hospital discharge (Table 2).

**Table 2:** Costs for patients with limited English proficiency versus controls.

Costs of hospital admission to hospital discharge			
Cohort	mean	95% CI	p-value
LEP	$54,494	$52,268 to $56,720	0.013
Control	$50,633	$48,565 to $52,7022	
Difference	$3,861	$822 to $6,900	
Costs of index ICU admission to hospital discharge			
Cohort	mean	95% CI	p-value
LEP	$50,696	$48,555 to $52,837	0.035
Control	$47,530	$45,525 to $49,538	
Difference	$3,166	$231 to $6,101	
Costs of all ICU admissions			
Cohort	mean	95% CI	p-value
LEP	$39,448	$37,495 to $41,402	0.226
Control	$37,778	$35,907 to $39,649	
Difference	$1,670	-$1,034 to $4,375	
Costs of post index ICU discharge to hospital discharge			
Cohort	mean	95%CI	p-value
LEP	$19,715	$18,019 to $21,411	0.121
Control	$17,903	$16,363 to $19,443	
Difference	$1,812	-$478 to $4,103	

### Survivors only propensity matched cohort

The propensity matched analysis of survivors included 4,694 in each group. The separate propensity score analysis among the patients that survived also found statistically greater total hospital costs in patients with LEP for all cost outcomes. The magnitude of the cost difference of LEP patients that survived was larger than that of the propensity score model using the full cohort. Additionally, all other cost outcomes showed greater costs among LEP patients in the survivors only propensity score model. Hospital admission to discharge—$4,208 (95% CI $1,517 to $6899, p = 0.002), index ICU admission to hospital discharge—$4,184 (95% CI $1,609 to $6758, p = 0,001), costs of all ICU admissions—$2,433 (95% CI $127 to $4739, p = 0.039) and costs of post index ICU discharge to hospital discharge—$3,034 (95% CI $1086 to $4,982, p = 0.002) (Table 3). We did not do propensity matched analysis of those that died.

**Table 3:** Survivors only propensity matched sample.

Costs of hospital admission to hospital discharge			
Cohort	Mean	95% CI	p-value
LEP	$51,400	$49,418 to $53,382	0.002
Control	$47,192	$45,372 to $49,012	
Difference	$4,208	$1,517 to $6,899	
Costs of index ICU admission to hospital discharge			
Cohort	Mean	95% CI	p-value
LEP	$48,196	$46,295 to $50,097	0.001
Control	$44,012	$42,276 to $45,749	
Difference	$4,184	$1,609 to $6,758	
Costs of all ICU admissions			
Cohort	Mean	95% CI	p-value
LEP	$36,591	$34,906 to $38,277	0.039
Control	$34,158	$32,585 to $35,732	
Difference	$2,433	$127 to $4,739	
Costs of post index ICU discharge to hospital discharge			
Cohort	Mean	95% CI	p-value
LEP	$18,751	$17,259 to $20,244	0.002
Control	$15,717	$14,467 to $16,968	
Difference	$3,034	$1,086 to $4,982	

### Stratified cohorts international versus resident

There were 1,142 international LEP patients. No matching was done with any analysis for international patients. Finally, analysis comparing costs of hospital admission to discharge between those with LEP who resided in the US ($52,396) versus international patients ($61,637) showed that international patients had on average $9,240 greater costs (95% CI $3341 to $15,140, p = 0.002;) than patients residing in the US. Cost comparisons of secondary cost outcomes of this subgroup analysis are provided in the S1 Table.

### Hospital and ICU Length of Stay (LOS)

The hospital LOS includes the complete hospitalization and any hospital days before admission to the ICU. Patients with LEP had statistically significant longer Hospital LOS than controls, However, there were no differences in ICU LOS (Table 4).

**Table 4:** Hospital and ICU LOS.

Hospital Length of Stay(days)			
Cohort	mean	95% CI	p-value
LEP	10.01	9.59 to 10.43	<0.001
Control	8.74	8.35 to 9.08	
Difference	1.30	0.73 to 1.86	
Total ICU Length of Stay(days)			
Cohort	mean	95% CI	p-value
LEP	3.65	3.38 to 3.92	0.075
Control	3.31	3.07 to 3.56	
Difference	0.33	-0.03 to 0.70	

## Discussion

The purpose of this study was to evaluate and compare the costs of critical illness including ICU and overall hospitalization between patients with LEP and patients who speak English (EP control). Due to substantial differences in the baseline characteristics of the LEP and EP groups, propensity score matching was used. Following propensity matching for the co-variates age, sex, race, ethnicity, APACHE III score, and Charlson Comorbidity score, we achieved the intended balance. We demonstrated that the costs for patients with LEP was significantly greater than controls for both the complete hospitalization and from index ICU admission to hospital discharge. Costs from post-ICU to hospital discharge and for all ICU admissions was not significantly different between those with LEP and controls. However, these results showed similar directionality to the significant cost findings. Additionally, when we did sensitivity analysis of those that survived, all outcomes were statistically significant, demonstrating greater costs for those with LEP. When we examined Hospital LOS, we noted those with LEP had Hospital LOS on average 1.3 days longer than controls and this likely contributed to costs.

Acknowledging that our institution treats a proportion of patients who travel internationally for care and that there may be challenges with transitions of care among these patients, we stratified the cohort to counter the potential that prolonged waits for transportation to native countries and challenges finding a rehab destination might influence overall outcomes for all patients with LEP [26]. Our analysis to examine differences in costs for international patients versus those who resided in the US determined that international patients with LEP had significantly greater costs than patients with LEP who were living in the US. This may be due to several factors, including acculturation and a familiarity with the US healthcare system among those who live in the US. Other considerations are cultural, religious and faith-based beliefs about healthcare utilization, health literacy, the influence of supporting local community, logistical challenges including lack of access to transitional care setting, and insurance and healthcare payment mechanisms [35].

Studies assessing cost for patients with LEP requiring critical care are limited and there is a dearth of studies examining cost effectiveness [36]. What is generally accepted is that ICU care does provide an improvement in survival and the cost per life saved falls for patients who have increased severity of illness [37]. ICU care can be considered as cost-effective as other “essential therapies”. These assessments are a broad generalization and may not apply in all cases and for all interventions. Costs are directly related to patient factors such as severity, acuity, and complexity of illness as well as chronic underlying co-morbidities. While we have found an association between increased costs and patients with LEP, we should also recognize that the fixed costs associated with ICU care, including buildings, equipment, salaried labor and overhead, are sizable and not directly influenced by patient factors [38]. Furthermore, those types of costs would apply to all patients.

There is much evidence to demonstrate that the use of interpreters improves the quality of clinical care for patients with LEP [39–42]. Many studies examining costs among patients with LEP focus on access to and utilization of interpreter services. When the role of interpreters in affecting costs is examined, studies tend to show no increase in overall costs and often cost savings by hospitals investing in interpreter services. However, the hospital setting in which interpreters are used may influence cost analysis conclusions [25, 43, 44]. Cost is also affected by interpreter modality, with phone and video interpretation being more feasible and affordable for many institutions [45]. What is likely, however, is that increased use of interpreters will improve communication and improve quality and timeliness of decision making, potentially influencing costs and almost certainly promoting care that is aligned with values and wishes [46]. Our own qualitative work indicates that differences in comfort care measure order use before imminent death among patients with LEP reflects both authentic preferences based on cultural and spiritual beliefs as well as sub-optimal bidirectional communication inhibiting decision making about care [47, 48].

In other healthcare settings, patients with LEP perceive that the care they receive is of poor quality, and there is much literature documenting poorer outcomes [12]. The relationship between increased costs and quality of care in the context of the ICU and hospital stay could not be assessed with this study. However, some studies suggest that there may be an inverse relationship between quality and costs and that palliative care interventions including high quality end of life conversations with physicians can significantly reduce healthcare costs and increase quality of care in the final week of life, especially among racial and ethnic minority groups [49]. However, patients with LEP may not be as likely to participate in high quality end of life conversations given potential barriers to accessing language services in a timely manner [50]. Time-limited trials and advance care planning were proposed by Curtis, et al. as approaches to improve quality of end of life care in the ICU as well as address cost concerns [51]. These mechanisms may be less acceptable to many patients with LEP based on evidence in the literature [52–54].

Strengths of this study include the following. This is the first study analyzing costs for patients with LEP who require critical illness care during a hospitalization. We analyzed data from a 10- year time frame and although we did not show statistically significant results for all secondary outcomes, directionality for increased costs among patients with LEP was apparent in all outcomes, supporting our hypothesis. Furthermore, although our institution is known for its management of complex care, including those patients who are sponsored by international governments to attend for care they cannot receive in their own countries, we were able to stratify patients with LEP to those who resided in the US and those who travelled from abroad. This sensitivity analysis provided granular information making our results more generalizable to centers which do not treat international patients with LEP.

Confounding factors such as race and ethnicity may sometimes limit interpretations about the specific role of language in outcomes. However, by utilizing propensity scoring we were able to successfully match our cohorts for these important variables, substantiating our findings. In addition, our propensity matching also accounted for APACHE III scores reflecting severity of illness as well as Charlson co- morbidity scores indicating concomitant chronic illness, other factors that might be more frequent in some populations. Our group has experience in conducting these types of analysis [22].

Our study has some limitations. This was a single center study conducted in a quaternary care academic center in the Midwest. Although we did sensitivity analysis using stratified data as described above, our patient population with LEP residing in the US may differ from most institutions in the US. Furthermore, although widely accepted in the literature, the definition of LEP has considerable limitations [55]. The definition “primary language other than English” as documented in the EMR does not necessarily reflect actual linguistic proficiency and may lead to misclassification of some patients [56]. Although decision makers in the context of ICU may often be family members, it is not feasible to assess or understand their language proficiency.

Additionally, this study did not examine whether interpreter services were used by the LEP patients but since we started this work, we have developed a method to identify when interpreters were used (rather than marked as needed or have a preferred language other than English). Future work will leverage this method to further understand when interpreters used and the impact on care and potentially cost. Therefore, we cannot evaluate the effect of interpreter use on costs as others have done. As in other institutions, interpreter use by clinicians in our institution is highly variable [57].

Furthermore, we did not account for health literacy in this analysis and that may compound the effect of language barriers [35]. Although we conducted propensity matched analysis of survivors, we did not do an analogous propensity matched analysis of those that died. Lastly, our study examined costs but did not assess cost effectiveness. We had initially intended to examine insurance status and its impact on cost but we were restricted by our institutional policy which does not allow the use insurance information for research and in publications.

Our approach to data abstraction meant our understanding of end-of-life decision making and specific procedures was not feasible. We did not abstract data about palliative care consults or types of end-of-life care. Our previous work has shown that those with LEP in the ICU use comfort measures less frequently and tend to delay the use of comfort care measures. They also have lower rates of do-not-resuscitate orders and tend to delay the use of this also when compared to patients who speak English [14, 47, 48, 53, 58, 59]. These factors could certainly contribute to hospitalization and healthcare utilization [14, 47, 48, 53, 58, 59]. Additionally, we could not bring to light humanistic aspects of micro-communication within the scope of this study. We could not assess who was making decisions including whether it was the patient or family members.

## Conclusion

This historical cohort study examined comparative costs over a 10-year period for patients with LEP experiencing a critical illness. We used propensity matching for potentially important confounders such as age, sex, race, ethnicity, APACHE III score, and Charlson Comorbidity score and demonstrated that the cost for patients with LEP was significantly greater than controls for both the complete hospitalization and from index ICU admission to hospital discharge. All other secondary outcomes showed a similar directionality of costs. The cause of these cost differences is not entirely clear. However, we believe improved communication can address some of these costs that may be related to suboptimal understanding of treatment options among families with LEP, and a default to continue or escalate care leading to increased healthcare utilization, use of interventions and prolonged ICU care.

## Supporting information

S1 Table Costs of international vs non-international LEP patients. (DOCX)

## Abbreviations

APACHE score: Acute Physiology and Chronic Health Evaluation score
EMR: electronic medical record
ICU: Intensive Care Unit
LEP: Limited English Proficiency
