# Supplementary material for: Comparative costs for critically ill patients with limited English proficiency versus English proficiency
Source: PLoS One. 2023 Apr 26;18(4):e0279126. doi: 10.1371/journal.pone.0279126 (PMC10132690; doi:10.1371/journal.pone.0279126)
Supplement: S1 Table — (DOCX) [file pone.0279126.s001.docx]

Supplemental Table. Comparison of international vs non-international LEP patients

| Costs of hospital admission to hospital discharge | | | | | | | | | |
| --- | --- | --- | --- | --- | --- | --- | --- | --- | --- |
| Cohort | Mean | | 95% CI | | | | | | p-value |
| International (n=1,142) | $61,637 | | $56,267 to $67,006 | | | | | | 0.002 |
| Non-international (n=3,981) | $52,396 | | $49,952 to $54,840 | | | | | |  |
| Difference | $9,240 | | $3,341 to $15,140 | | | | | |  |
| Costs of index ICU admission to hospital discharge | | | | | | | | | |
| Cohort | Mean | | | 95% CI | | | | | p-value |
| International (n=1,142) | $56,571 | | | $51,442 to $61,700 | | | | | 0.008 |
| Non-international (n=3,981) | $48,960 | | | $46,583 to $51,337 | | | | |  |
| Difference | $7,611 | | | $1,958 to $13,264 | | | | |  |
| Costs of all ICU admissions | | | | | | | | | |
| Cohort | | Mean | | | 95% CI | | p-value | | |
| International (n=1,142) | | $40,943 | | | $36,534 to $45,351 | | 0.437 | | |
| Non-international (n=3,981) | | $38,982 | | | $36,735 to $41,230 | |  |  |  |
| Difference | | $1,960 | | | -$2,988 to $6,909 | |  | | |
| Costs of post index ICU discharge to hospital discharge | | | | | | | | | |
| Cohort | | Mean | | | | 95% CI | | p-value | |
| International (n=1,142) | | $25,415 | | | | $21,063 to $29,768 | | 0.002 | |
| Non-international (n=3,981) | | $18,069 | | | | $16,412 to $19,726 | |  |  |
| Difference | | $7,346 | | | | $2,689 to $12,003 | |  | |
